# Measurement of the nociceptive flexion reflex threshold in critically ill patients – a randomized observational pilot study

**DOI:** 10.1186/s12871-021-01490-8

**Published:** 2021-11-05

**Authors:** Benedikt Schick, Benjamin Mayer, Steffen Walter, Sascha Gruss, Ronald Stitz, Pauline Stitz, Eberhard Barth

**Affiliations:** 1 Department of Anesthesiology and Intensive Care Medicine, University Hospital Ulm, Albert-Einstein-Allee 23, 89081 Ulm, Germany; 2 Institute of Epidemiology and Medical Biometry, Ulm University, Schwabstraße 13, 89075 Ulm, Germany; 3 Department of Medical Psychology, Ulm University, Frauensteige 6, 89075 Ulm, Germany

**Keywords:** Pain, Critically ill patient, Nociceptive flexion reflex threshold, Behavioral Pain Scale, Excessive analgesia

## Abstract

**Background:**

Pain detection and treatment is a major challenge in the care of critically ill patients, rendered more complex by the need to take into consideration the risk of insufficient or excessive analgesia. The nociceptive flexion reflex threshold (NFRT) has become the established basis for measuring the level of analgesia in the perioperative context. However, it remains unclear whether NFRT measurement can be usefully applied to mechanically ventilated, analgosedated critically ill patients who are unable to communicate. Therefore, the aim of the present study was to investigate whether there is an association between the NFRT measurement and the Behavioral Pain Scale (BPS) in critically ill, analgosedated, and mechanically ventilated patients and whether the NFRT measurement can also detect potential excessive analgesia.

**Methods:**

This prospective, observational, randomized single-center pilot study included patients admitted to the surgical Intensive Care Unit of University Hospital Ulm, Germany, all of whom were analgosedated and intubated. Major exclusion criteria were defined as the need for the administration of neuromuscular blocking agents or neurological diseases associated with peripheral nerve conduction restriction. Initial NFRT and BPS measurements were conducted within 12 h after admission. A structured pain assessment was performed at least twice daily until extubation throughout the observation period thereafter (Group A: BPS + NFRT, Group B: BPS).

**Results:**

114 patients were included in the study. NFRT is associated negatively with BPS. NFRT was almost twice as high in patients with a Richmond Agitation Sedation Scale (RASS) score of -5 than in patients with a RASS score ≥ -4 (RASS -5 – NFRT: 59.40 vs. RASS -4 – NFRT: 29.00, p < 0.001).

**Conclusions:**

NFRT measurement is associated negatively with the BPS in critically ill patients. NFRT measurement provides guidance for the evaluation of nociceptive processes in patients with RASS scores ≤ −4, in whom analgesia level is often difficult to assess. However, in order to identify excessive analgesia and derive therapeutic consequences, it is necessary to gradually decrease analgesics and sedatives until a stimulus threshold is reached at which the patient does not feel pain.

**Trial Registration:**

Retrospectively registered in the German Clinical Trials Register, registration number DRKS00021149, date of registration: March 26, 2020. https://www.drks.de/drks_web/navigate.do?navigationId=trial.HTML&TRIAL_ID=DRKS00021149.

**Supplementary Information:**

The online version contains supplementary material available at 10.1186/s12871-021-01490-8.

## Background

 According to the International Association for the Study of Pain (IASP), Williams et al. describe pain as a “distressing experience associated with actual or potential tissue damage with sensory, emotional, cognitive and social components” [1]. Although the conscious perception of pain, especially the emotional aspect, is largely suppressed under general anesthesia [2, 3], nociception, i.e., pain encoding and processing, occurs constantly – even under deep anesthesia [4].

Pain is not only important in the perioperative context, but is also of great significance in critically ill patients. Adequate pain management for critically ill patients is still a challenge, even in modern intensive care medicine. Mechanically ventilated patients in particular are at risk of excessive analgesia [5, 6]. The importance of appropriate analgesia and minimal sedation is addressed in various guidelines and also by the eCASH (“early Comfort using Analgesia, minimal Sedatives and maximal Humane care”) approach introduced by Vincent et al. [7–9].

Analgesia and sedation during general anesthesia are broadly similar in terms of dosage and drug type and differ from profound analgesia, which is sometimes required in mechanically ventilated, critically ill patients.

At present, pain assessment in profoundly analgosedated, critically ill patients who are not able to self-assess their pain, e.g., using the Numeric Rating Scale (NRS), is predominantly limited to the interpretation of physiological parameters. The Behavioral Pain Scale (BPS) [10] is acknowledged as an effective scoring system for assessing pain. The BPS interprets pain based on a patient’s adaptation to ventilation, grimacing and upper limb movement. However, the interpretation of pain based on subjective criteria is open to misinterpretation [7]. Established pain assessment tools reach their limits in certain situations, especially in patients requiring high dosages of analgesics and sedatives [10–12].

Measurement of the RIII-reflex threshold of the nociceptive flexor reflex (NFR) is a particularly interesting option for addressing the limitations of pain assessment in critically ill patients who are unable to communicate. “The reflex response of the nociceptive flexion reflex consists of three components. The RIII reflex component can be recorded 90 ms to 150 ms after a painful stimulus, which is mediated by Aδ- and C-fibers. The reflex responses preceding the RIII component are mediated primarily by fast-conducting Aβ fibers and correspond to the reflex response to non-painful stimuli.“ [13, 14]. In patients under general anesthesia, measurement of the NFR-threshold (NFRT) has been shown to correlate negatively with the response rate to a painful stimulus [15–18]. To date, there is a lack of systematic studies investigating the NFR measurement in critically ill, mechanically ventilated patients who are unable to communicate.

Another equally important aspect in the context of pain assessment in critically ill patients is the quantification of adequate and appropriate analgesia. Analgesia is usually administered empirically, especially in profoundly sedated patients. The empirical approach carries the risk of excessive opioid use with adverse side effects such as respiratory depression, constipation, ileus, and pruritus [19].

Therefore, the aim of the present pilot study was to examine whether measuring the stimulus threshold of the nociceptive flexion reflex can be a useful complement to pain assessment using the BPS.

## Methods

### Study design

The present study was designed as a prospective, monocentric, randomized, observational pilot study. It was approved by the ethics committee of the University of Ulm, Germany (Trial Code No. 284/18) and registered retrospectively in the German Clinical Trials Register (DRKS ID: DRKS00021149). This study adhered to the STROBE (STrengthening the Reporting of OBservational studies in Epidemiology) principles and was conducted between November 2018 and March 2020 in the interdisciplinary 12-bed surgical intensive care unit (ICU) at Ulm University Hospital, Germany. Cardiosurgical patients were treated in a different ICU and were only transferred to the interdisciplinary ICU in the event of capacity issues. Data were evaluated between 4/2020 and 11/2020.

### Pain assessment – Measurement of the nociceptive flexion reflex threshold

**Fig. 1:**
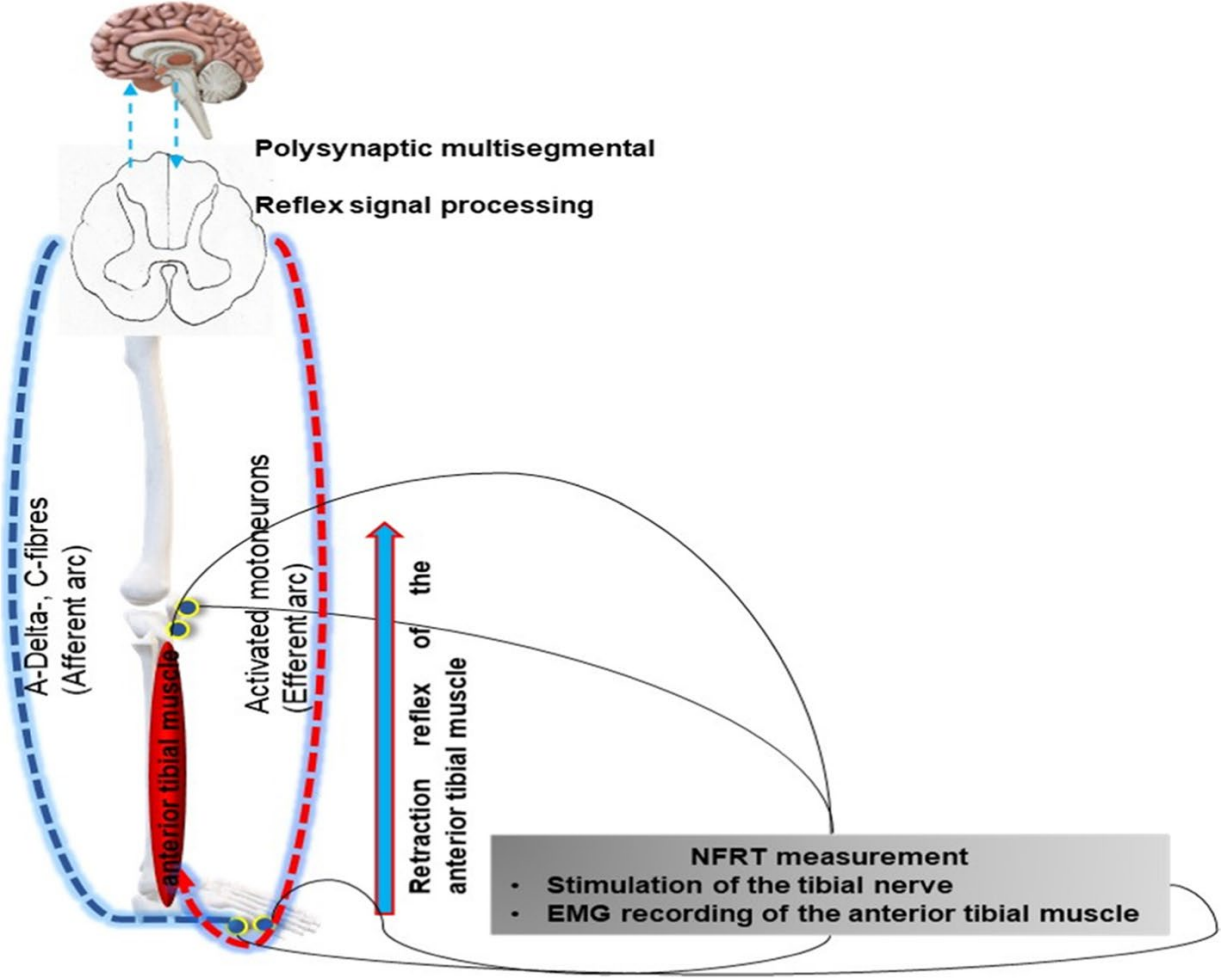
Schematic diagram of the measurement of the nociceptive flexion reflex threshold (NFRT)

Figure 1 schematically illustrates the measurement of the NFR stimulus threshold.

The NFRT, which is determined using the Paintracker® device, essentially examines the excitability of the spinal component of the pain processing system. The afferent reflex arc is composed of A-delta and C-fibers. Electrodes on the medial plantar surface are used to stimulate the tibial nerve. The stimulated A-delta and C-fibers conduct the stimulus to the spinal cord, where the polysynaptic circuitry of the stimulus occurs. This, in turn, causes motor neuron activation. The activated motoneurons represent the efferent element of the reflex. The resulting stimulus response can be recorded via a surface electromyogram on the tibialis anterior muscle. A reduction in the excitability of the reflex arc is reflected as an increase in the reflex threshold. The threshold tracking, i.e., the automated determination of the reflex threshold, is calculated by delivering five single rectangular pulses, each lasting 1 ms duration, with a 4 ms interpulse interval (200 Hz). The stimulation threshold is determined by automatically varying the intensity of the stimulation current. This correlates with the subjective pain threshold in patients who are awake [14–16, 20, 21]. Figure 1 was created by B. Schick©.

The interpretation of stimulus thresholds in different patients is critical because different surgical procedures are associated with different levels of pain intensity. For example, thoracic procedures are very painful and often require an epidural catheter technique for pain management. By contrast, patients with cerebral hemorrhage often have impaired central pain processing. NFRT measurement is particularly suitable for these patients, since this method records nociception, i.e., pain processing, rather than the subjective perception of “pain.“

### Study participants

 The study protocol was in accordance with the Declaration of Helsinki ethical guidelines. All patients or their legal representatives signed written informed consent forms to take part in this study. Inclusion criteria were: (a) age ≥ 18 years, (b) need for intensive care treatment and intubation, (c) expected ICU stay of at least 24 h, (d) written informed consent of the patient’s legal representative and post-hoc verbal and written patient consent. Patients were excluded from the study based on the following criteria: (a) age < 18 years, (b) pregnancy, (c) neurological diseases associated with a restriction of peripheral nerve conduction, (d) pacemaker/implanted cardioverter defibrillator, (e) need for neuromuscular blocking agent, (f) local anesthesia and epidural analgesia.

### Patient recruitment

Prior to the first NFRT measurement, the anesthesia documentation was used for all patients admitted to the ICU to verify complete neuromuscular recovery by means of train-of-four (TOF) monitoring at the end of surgery (TOF ratio > 0.9). In emergency cases, we checked whether the patient had been given a muscle relaxant by the emergency physician. Similarly, it was ensured that neither neuromuscular blockade due to local anesthetics nor epidural anesthesia was present. Spontaneous activity of all extremities was observed in neurosurgical patients/patients with intracranial hemorrhage during the daily wake-up test to exclude the possibility that the motor response to the nociceptive stimulus was impaired.

Potentially eligible patients were first evaluated on the basis of the inclusion and exclusion criteria. Then, simple, unrestricted randomization was performed by means of a random table generated using “Research Randomizer.“ Patients assigned even numbers were randomized to the group in which both BPS and NFRT were measured, referred to as Group A. Patients who were assigned odd numbers were randomized to the group where only BPS was measured, termed Group B.

Since NFRT measurement was not part of routine clinical practice in the group of patients investigated, a comparison group was randomized by means of simple randomization in order to verify whether the measurement of the NFRT had an impact on the outcome or analgesic management of ICU patients. Due to the lack of evidence in this regard, the NFRT measurement in the present study was intended to be observational only rather than interventional.

A structured pain assessment was conducted for all study patients within 12 h of admission to the intensive care unit. Pain assessments were conducted by two trained medical students throughout the entire study period.

Both BPS and NFRT measurements were performed for Group A patients. In Group B, pain levels were measured using BPS only. During the subsequent observation period, a structured pain assessment was conducted at least twice daily until extubation (Group A: BPS + NFRT, Group B: BPS).

BPS and NFRT were measured in all patients at rest with a minimum interval of 30 min from the previous nursing or medical intervention. Repetitive measurements were performed whenever possible and are included in the data analysis. This explains the difference between the number of patients measured and the number of NFRT measurements as well as the difference in the number of BPS and RASS measurements.

### Analgesia and sedation

#### Analgesics

Patients received sufentanil (µg/h) or remifentanil (mg/h) as continuous intravenous infusions. The analgesic regimen was adapted according to the BPS scale with the aim of achieving BPS scores of 3–4 (mild pain). Remifentanil dosage was administered in the ICU in mg/h via a perfusor, in contrast to the dosing specification generally applied for intraoperative use (µg/kg body weight).

During surgical procedures, metamizole was administered as a continuous infusion unless there were any contraindications. Metamizole is a prescription analgesic frequently used in the perioperative context in Germany, the mechanism of action of which is not yet fully understood. As a prodrug, it is hydrolyzed to the active N-methylaminoantipyrine and acts both centrally and peripherally. Indications include mild to moderate pain, postoperative pain, and tumor pain. The clinical duration of action is 4 to 6 h. The daily dosage is calculated as 10 to 15 mg/kg, with a maximum dose of 60 mg/kg [22]. Generally, adults are given 500 to 1000 mg intravenously every 6 h or a dose of 4 g per 24 h (continuous use) as postoperative analgesia. Metamizole is not approved in some countries because of potential adverse drug reactions, which include risk of agranulocytosis and increased risk of severe allergic reactions.

When a patient was already less deeply analgosedated (RASS ≥ -3), the opioid piritramide was administered intravenously to treat anticipated painful stimuli such as patient positioning. “As a 4-aminopiperidine derivative, it is a pure µ-agonist and the opioid most commonly used for postoperative analgesia in Germany. The potency is approximately 0.7 times that of parenteral morphine. The dosage is generally 0.1 mg/kg or 3.5 – 7.5 mg as an intravenous bolus. The clinical context half-time is 4 to 10 hours. Metabolization is almost entirely hepatic” [22].

### Hypnotics

With regard to intravenous hypnotics, patients received either propofol (mg/h) or lormetazepam (mg/h) as a continuous infusion. The dosage was adjusted with respect to the previously determined RASS score.

### Study objectives

The objectives of the study were as follows:

- Is there a correlation between the NFRT and the BPS and the Richmond Agitation Sedation scale in critically ill, mechanically ventilated, analgosedated patients who are unable to communicate in Group A?
- Do the measured stimulus thresholds for the nociceptive flexion reflex differ between the various specialties in patients in Group A?
- Does NFRT measurement detect potential excessive analgesia in critically ill, analgosedated, mechanically ventilated patients who are unable to communicate in Group A?
- Measurement of a cohort of critically ill patients in whom pain is assessed using only the BPS (Group B) in order to demonstrate that Cohort A patients were not selected arbitrarily in favor of the measurement method.

### Data analysis

#### Sample size calculation and power analysis

A sample size of 105 patients per group was calculated using GPower 3.1 to achieve a power of 80 % with a two-sided α-level < 0.05 and an effect size of d = 0.5.

### Statistical analysis

The following patient-related data were collected during patient stays in the ICU: (a) age upon registration & sex, (b) primary reason for ICU admission & length of ICU stay, (c) BPS & Richmond Agitation Sedation Scale (RASS), (d) Sequential Organ Failure Assessment Score (SOFA-Score), (e) Therapeutic Intervention Scoring System (TISS-28) and Simplified Acute Physiology Score (SAPS II), (f) vital signs, (g) analgesics and hypnotics. Data were collected in Microsoft EXCEL 2010® (Microsoft Corp., Redmond, WA) and analyzed using Sigma Plot Version 14® for Windows (Systat Software GmbH, Erkrath, Germany) and SAS Version 9.4 (SAS Institute GmbH, Heidelberg, Germany). Quantitative data were expressed as median, minimum and maximum values and were compared for nonparametric distributions using the Wilcoxon Matched Pairs test. In the analysis of the independent samples, the Student’s t-test was used for normally distributed data (testing by Shapiro-Wilk). In the absence of normal distribution, the Mann-Whitney rank-sum test was performed. Due to the early cessation of the trial, all results reported must be interpreted in an exploratory manner. Thus, adjustment of the p-values for multiple testing was not required. A linear model (LM) was applied to evaluate possible associations between the measured NFRT and the key baseline characteristics which were not assessed in a time-dependent manner. We also used mixed linear regression modelling (MLM) to assess the possible association between NFRT and BPS measurements, which enabled us to account for the repeated-measures structure of the data. Specifically, the time of measurement was added as a further independent predictor alongside the BPS values. The repeated-measures structure was implemented by means of a random intercept. An explorative, two-sided type 1 error level of 5 % was applied to all analyses.

## Results

**Fig. 2:**
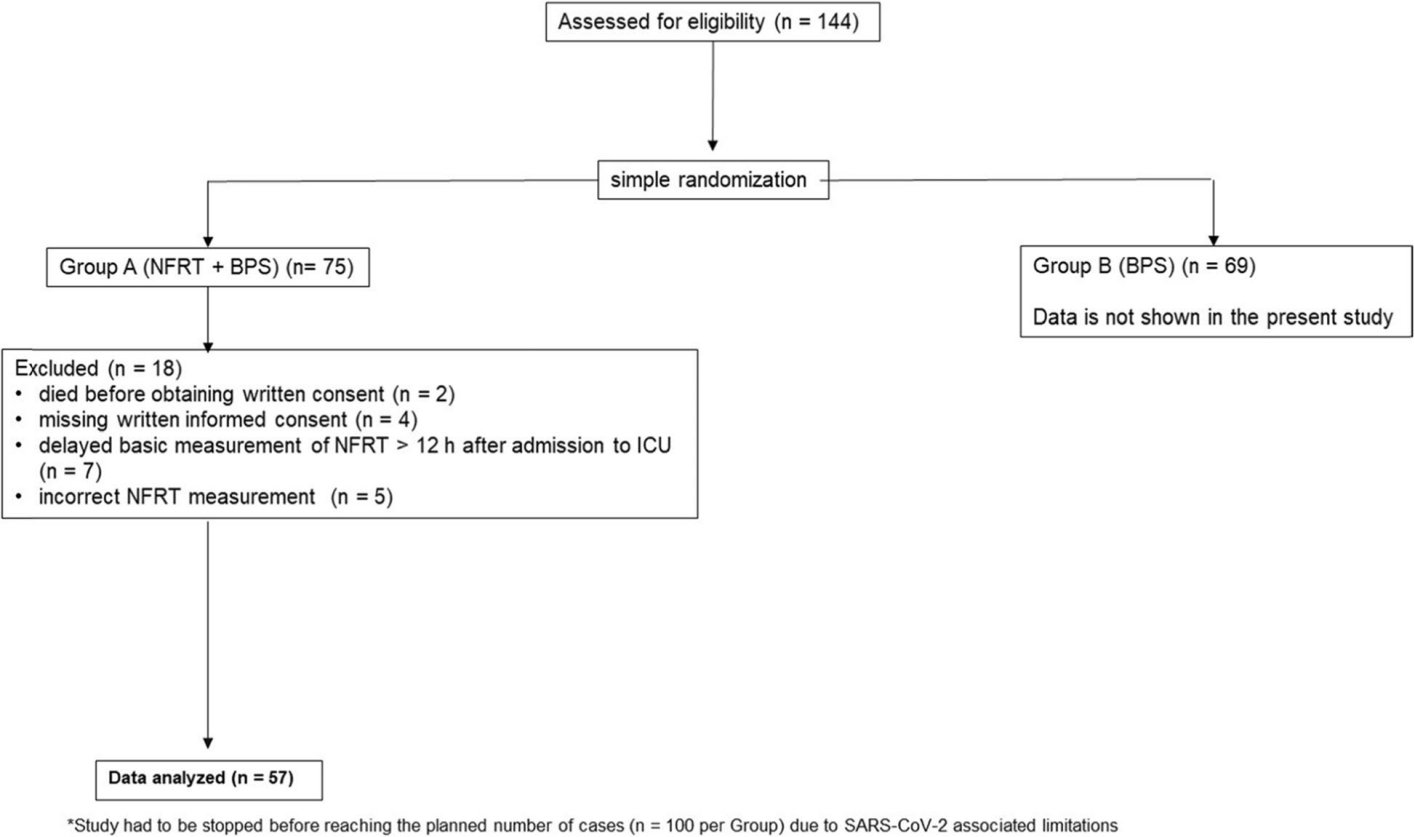
Study flow chart

All eligible patients were assessed based on the inclusion and exclusion criteria and then divided into two groups using simple randomization (Fig. 2). The characteristics of both groups are described in detail in Table 1.

**Table 1:** Patient characteristics

Variable	Patients n =114		
	Group A BPS-NFRT n = 57	Group B BPS n = 57	P
**Age**			
Median *	61.0 (54.0 – 72.0)	68.0 (63.0 – 81.0)	<0.001
**Sex, n (%)**			
Male	44 (77.2)	37 (64.9)	0.221
Female	13 (22.8)	20 (35.1)	0.070
**ICU** – **LOS**			
Median *	10.0 (5.0 – 19.0)	10.0 (5.0 – 20.5)	0.756
**Mortality, n (%)**	6.0 (10.5)	15.0 (26.3)	0.029
**Disease severity scoring**			
SAPS II *	36.0 (29.0 – 45.0)	38.0 (32.0 – 47.0)	0.374
TISS–28 *	19.0 (14.0 – 24.0)	18.0 (10.0 – 24.0)	0.285
**Analgesics and sedatives**			
Sufentanil μg/h			
Median *	12.5 (5.6 – 20.0)	15.0 (5.0 – 20.0)	0.388
n (%)	8 (14.0)	7 (12.3)	0.68
Remifentanil mg/h			
Median *	0.3 (0.2 – 0.4)	0.2 (0.2 – 0.3)	<0.001
n (%)	37 (64.9)	37 (64.9)	1.000
Metamizole mg/h			
Median *	168.0 (168.0 – 168.0)	168.0 (168.0 – 168.0)	0.167
n (%)	36 (63.2)	39 (69.4)	0.570
Propofol mg/h			
Median *	200.0(140.0 – 200.0)	200.0(100.0 – 280.0)	1.000
n (%)	41 (71.9)	37 (64.9)	0.462
Lormetazepam mg/h			
Median *	0.6 (0.3 – 0.8)	0.6 (0.5 – 0.8)	0.801
n (%)	12 (21.1)	13 (22.8)	0.74
**Measurement of sedation depth and pain intensity**			
Richmond Agitation Sedation Scale – Median *			
	-4.0 (-5.0 – -3.0)	-3.0 (-4.0 – -2.0)	0.001
Behavioral Pain Scale			
Median *	3.0 (3.0 – 3.0)	3.0 (3.0 – 4.0)	0.631
BPS 3 (n)	51	56	0.875
BPS 4 (n)	28	57	<0.001
BPS 5 (n)	6	4	0.38
BPS 6 (n)	2	1	n.e.
BPS 7 (n)	1	0	n.e.
BPS 8 (n)	1	0	n.e.
BPS 9 (n)	1	0	n.e.
**Primary reason for ICU admission, n (%)**			
Neurosurgery & brain hemorrhage	13 (22.8)	16 (28.1)	0.37
Abdominal surgery	15 (26.3)	15 (26.3)	1.00
Trauma surgery	5 (8.8)	6 (10.5)	0.65
Cardiac surgery	2 (3.5)	1 (1.8)	-----------
Vascular surgery	4 (7.0)	8 (14.0)	0.16
Thoracic surgery	5 (8.8)	3 (5.3)	0.37
Respiratory failure	5 (8.8)	3 (5.3)	0.37
Internal medicine	-----------	1 (1.8)	-----------
Urology	7 (12.3)	4 (7.7)	0.24
Oral and maxillofacial surgery	1 (1.8)	-----------	-----------

Note: The second column indicates the group in which both BPS and NFR were measured, while the third column shows the patients in whom only the BPS score was recorded. Data are shown as median* values (interquartile range) or numbers (percentage). Rounding errors led to a total percentage > 100%. Differences between groups were determined using Student's t-test (Shapiro-Wilk normality test passed) or the Mann-Whitney U-test (normality test failed); P-values are not adjusted for multiple testing. Abbreviations: ICU: Intensive Care Unit, SAPS II: Simplified Acute Physiology Score II, TISS-28: Therapeutic Intervention Scoring System 28, CAM-ICU: Confusion Assessment Method for the Intensive Care Unit. n.e. = not established due to the small number of patients

### Patient characteristics

#### Univariate Analysis

Univariate LM analyses were performed to determine whether the NFRTs measured were affected by demographic factors such as age, sex, body height and weight, and length of ICU stay. We did not find that any of the aforementioned determinants had a statistically significant influence on the NFRT measurement, as shown in Table 1 in the Additional file 1.

We then investigated whether there was any association between the measured NFRTs and the BPS scores assessed for patients in Group A.

### Association between NFRT and BPS

**Fig. 3:**
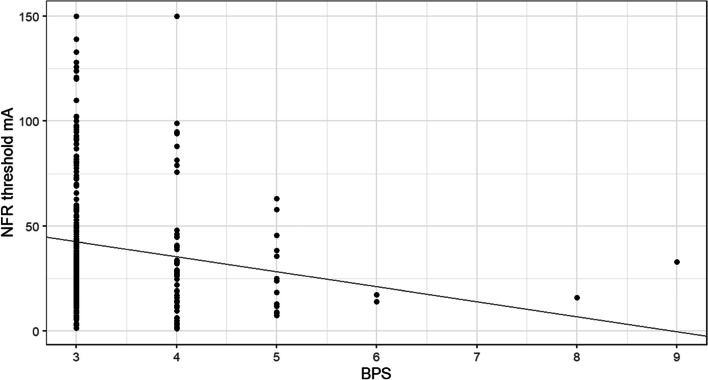
Mixed model calculation with NFRT as the target variable and BPS as the influencing variable, corrected for repeated-measures structure. The black line indicates the regression line corrected for repeated measures

With a total of 297 measurements in 57 patients, the NFRT was defined as the dependent variable and the BPS as the independent predictor in a mixed model. Corrections were made for the repeated-measures structure. The resulting regression line, which takes into account the data dependency effect, indicates that there is a negative association between the BPS and the NFRT (Fig. 3).

### Difference between NFRTs at various BPS scores

**Fig. 4:**
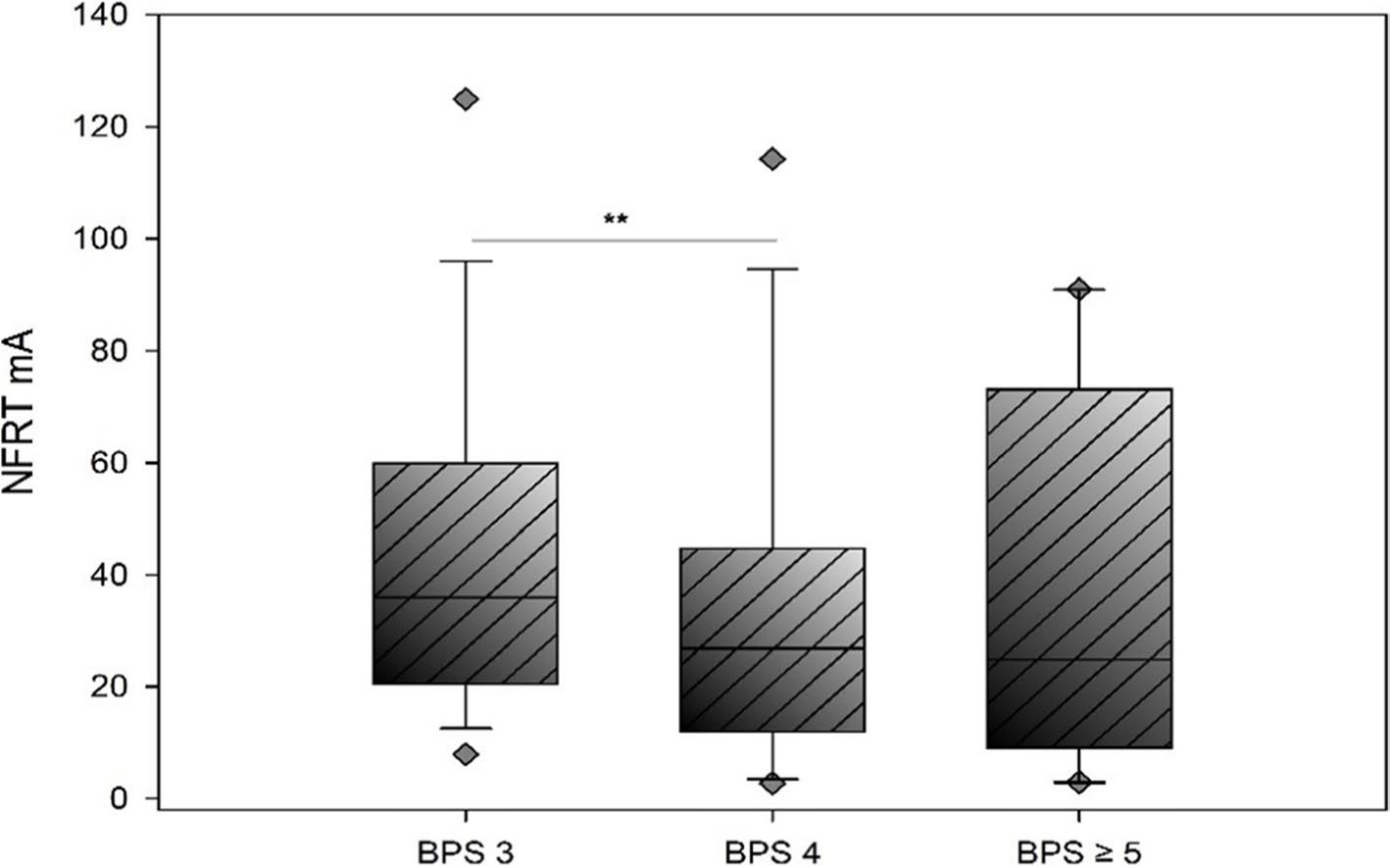
Comparison of the NFR thresholds for Group A patients with BPS scores of 3 compared to BPS scores of 4 and BPS scores ≥ 5. Statistically significant differences between NFR thresholds of BPS 3 and 4 (p-value = 0.005) were calculated using a Mann-Whitney rank-sum test. No statistically significant differences were found between NFR thresholds of BPS 3 and BPS 4 and BPS ≥ 5 (p = 0.178, p = 0.959). Number of patients (n_p_) and measurements (n_m_) per group: BPS 3: n_p_ = 51, n_m_ = 210, BPS 4: n_p_ = 28, n_m_ =53, BPS ≥ 5: n_p_ = 8, n_m_ = 15)

Figure 4 depicts the results of the NFRT measurements in relation to the respective BPS score from 3 to ≥ 5 among patients in Group A. Patients whose NFRT was measured at a BPS score of 3, which is classified as free of pain, have a median threshold of 36.00 mA (n = 51 patients, 210 measurements, median threshold 36.00 mA, IQR = 20.50 – 60.00 mA). At a BPS score of 4, corresponding to slight pain, a median NFRT of 26.90 mA (IQR = 16.85 – 52.3 mA, measurements = 53) was determined in 28 patients. A comparison of the stimulus thresholds measured for BPS 3 and BPS 4 indicates that patients with an assumed absence of pain tended to have a higher NFRT than patients with mild pain (Mann-Whitney rank-sum test – p-value: 0.005) to a statistically significant extent. Because of the wide confidence intervals, however, these results should be interpreted cautiously and taking into consideration the patient’s clinical condition, despite their statistical significance. There is no statistically significant difference with respect to the NFRTs measured at a BPS ≥ 5 (median NFRT = 24.90 mA, IQR = 9.10 – 73.00 mA, n = 11, measurements = 15, Mann-Whitney rank- sum test, p-value: 0.178 (BPS 3 vs. BPS ≥ 5), p = 0.959 (BPS 4 vs. BPS ≥ 5). The results are displayed in Fig. 4.

**Fig. 5:**
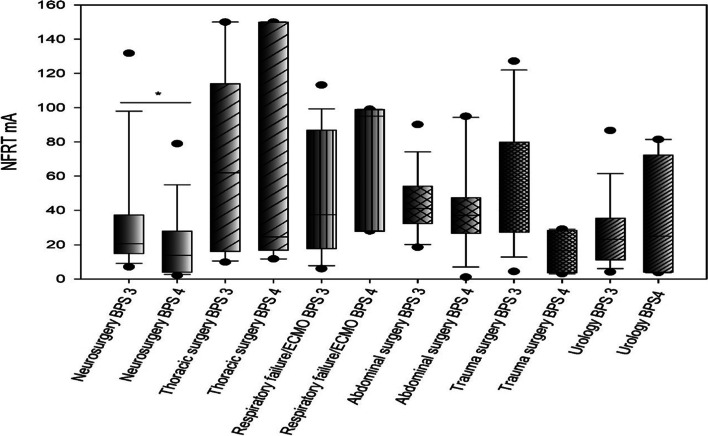
Comparison of NFRT in patients with BPS scores of 3 and 4 for the different specialties

A graphical analysis was performed to assign the determined stimulus thresholds in patients with BPS scores of 3 and 4 to the different operations/diseases, (Fig. 5 and Additional file 1: Table 2). Neurosurgical patients/patients with intracranial hemorrhage and a BPS score of 3 had significantly higher stimulus thresholds than neurosurgical patients with a BPS score of 4. Stimulus thresholds differed among medical specialties, sometimes significantly. Figure 5 emphasizes the fact that different NFRTs are measured in patients with different surgical procedures and diseases despite comparable pain states (BPS 3 and 4). This in turn reflects the different analgesic needs of patients. Neurosurgical procedures or interventions on the brain itself are usually only slightly painful whereas thoracic or abdominal procedures, for example, are sometimes associated with severe pain and therefore require intensified pain therapy.

Patients with neurosurgical intervention/intracranial hemorrhage had a statistically significantly higher NFRT at BPS 3 than at BPS 4 (Mann-Whitney U test: p = 0.042, median NFRT BP 3: 20.50 mA, IQR: 15.02 − 37.30 mA vs. median NFRT at BPS 4: 13.75 mA, IQR: 4.0 − 28.0 mA). Patients undergoing thoracic surgery had the highest NFRT at BPS 3 with 62.0 mA, IQR: 16.3 − 114.0 mA. Neurosurgical patients had the lowest NFRT at BPS 3 (median NFRT BPS 3: 20.50 mA, IQR: 15.02 − 37.30). Similarly, the highest NFRT at BPS 4 for patients with respiratory failure/ECMO was 92. 0 mA, (IQR: 28.0 − 99.0). The lowest NFRT at BPS 4 was recorded in patients with neurosurgery intervention/intracranial hemorrhage with 13.75 mA (IQR: 4.0 − 28.0 mA). For details of the NFRTs corresponding to the BPS scores, please see Additional file 1: Table 2.

The amounts of analgesics administered in Group A patients in comparable pain conditions were analyzed with respect to the different stimulus thresholds resulting from different surgeries and diseases (Table 2). Patients received the highest amount of remifentanil after trauma surgery. Nevertheless, despite high analgesic requirements, the median NFRT was not highest in the trauma group. Thus, there is no linear relationship between BPS, NFRT, and the amount of analgesics administered. Patients with respiratory failure/ECMO therapy received the most sufentanil during the observation period, which was reflected in a high NFRT. It is possible that the sedative effect of sufentanil was exploited in these cases to provide deep analgesia for patients with severe ARDS. The analgesic requirements of this patient group appear to be rather low.

**Table 2:** Summary of remifentanil and sufentanil doses in patients with BPS 3 and 4

Specialty	BPS	NFRT _**Remifentanil**_ * [mA]	Remifentanil* [mg/h]	NFRT _Sufentanil_^*^ [mA]	Sufentanil* [μg/h]
Neurosurgery/ brain hemorrhage	3	19.20 [15.00 – 39.45]	0.20 [0.20 – 0.30]	30.50 [19.57 – 37.75]	10.00 [10.00 – 13.12]
Abdominal surgery	3	42.50 [33.90 – 58.30]	0.30 [0.23 – 0.40]	41.80 [25.75 – 72.00]	10.00 [0.01 – 10.00]
Trauma surgery	3	25.00 [15.30 – 33.75]	0.60 [0.40 – 0.60]	61.43 [34.03 – 87.08]	15,00 [15.00 – 20.00]
Thoracic surgery	3	22.25 [11.95 – 35.27]	0.20 [0.10 – 0.20]		
Respiratory failure	3	35.5 [18.89 – 42.65]	0.20 [0.20 – 0.20]	89.00 [82.50 – 93.25]	20.00 [18.75 – 20.00]
Urology	3	17.00 [11.50 – 34.20]	0.30 [0.30 – 0.40]		
Neurosurgery/brain hemorrhage	4	n.e.	n.e.		
Trauma surgery	4	n.e.	n.e.		
Respiratory failure	4	n.e.	n.e.		
Urology	4	n.e.	n.e.		
Thoracic surgery	4	18.90 [14.30 – 50.10]	0,30 [0.100 – 0.30]		
Abdominal surgery	4	40.50 [29.52 – 77.50]	0.30 [0.21 – 0.40]		

* Median, IQR n.e. = not established, too few individual values to calculate the descriptive statistics

The postoperative dosage of metamizole was almost the same in Group A and Group B. Here, the dose of 168 mg/h is equivalent to a continuous infusion rate of 2.1 ml/h at a standard dosage of 4 g metamizole to 50 ml NaCl 0.9 %. Metamizole was administered postoperatively in both groups. Piritramide was administered in a small number of patients in both groups as a single dose before a nursing intervention or planned wake-up attempt.

#### NFRT at different RASS values

**Fig. 6:**
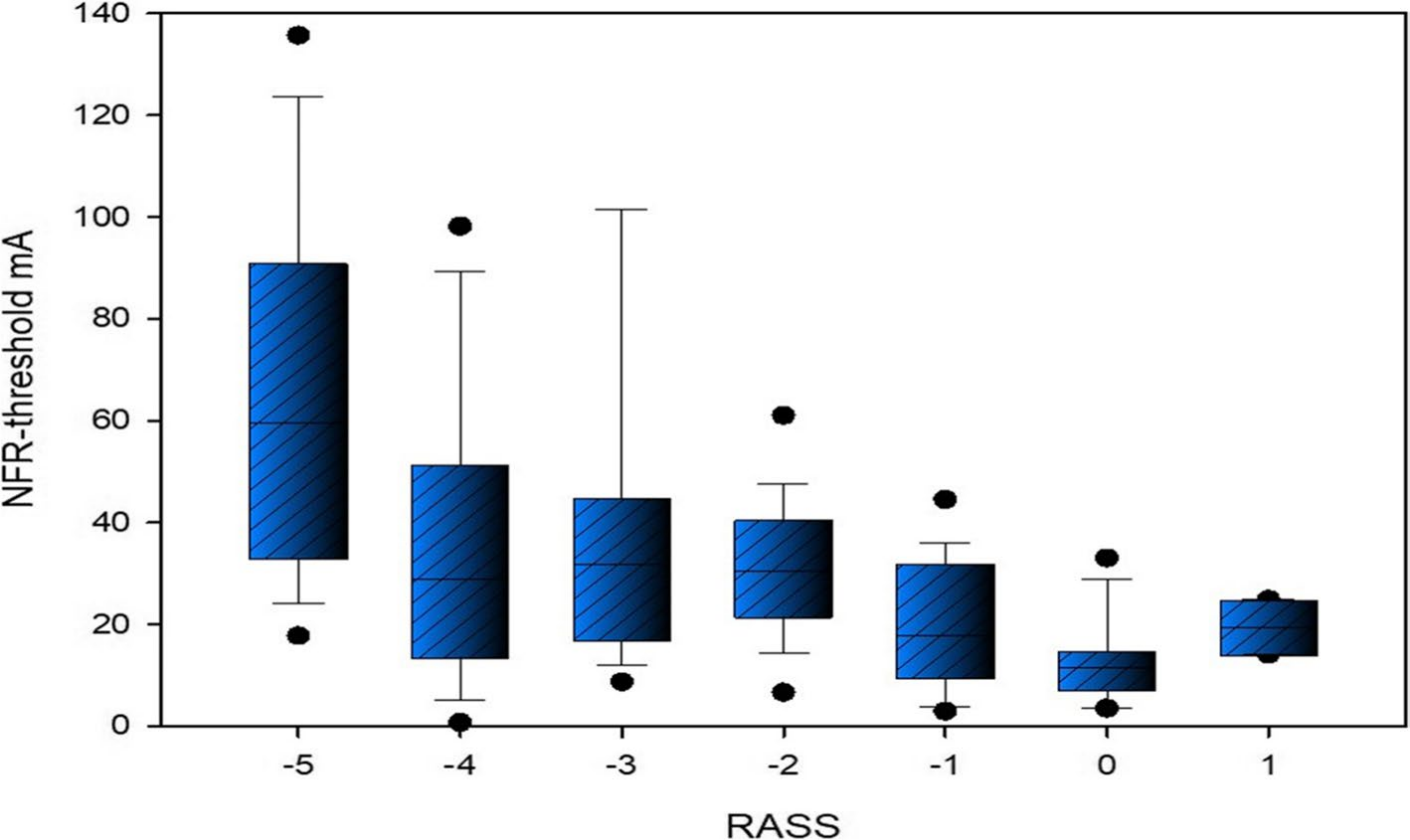
NFRT at different RASS values. The statistical differences in the median NFRT with respect to the respective RASS value were calculated using Mann-Whitney rank sum tests or Welch´s t-tests. The statistical results are shown in detail in Table 3 in the Additional file 1. RASS = Richmond Agitation Sedation Scale, NFR = nociceptive flexion reflex, IQR = interquartile range

Comparing the measured median NFRTs at different RASS values, it can be seen that the deeper the sedation, the higher the corresponding stimulus threshold. This is depicted in Fig. 6 and in Table 3 in the Additional file 1.

Patients with a RASS-Score of -5 have a median stimulus threshold of 59.40 mA (IQR: 32.95 – 91.00) which is higher to a statistically significant extent than less deeply sedated patients. (Mann-Whitney rank sum test, RASS -4 = 29.00 mA, IQR = 13.56 – 51.52, p < 0.001, RASS -3 = 31.75 mA IQR = 16.88 – 44.92, p < 0.001, RASS -2 = 30.50 mA, IQR = 21.50 – 40.63, RASS -1 = 17.90 mA, IQR = 9.50 – 32.00, p < 0.001, RASS 0 = 11.50 mA, IQR = 7.05 – 14.80, p < 0.001, RASS 1 = 19.50 mA, IQR = 14.10 – 24.90, p = 0.032).

These results indicate that very deeply sedated patients are at a high risk of excessive analgesia. Stimulus thresholds of > 90 mA appear to be too high considering that patients sedated to a RASS of -4 showed much lower peak stimulus thresholds.

Due to the outbreak of the SARS-CoV-2 pandemic in spring 2020, we decided to terminate the study prematurely with the current number of cases. Comparable studies include a patient population of between 40 and 100 patients [21, 23]. Nevertheless, we performed a post-hoc power analysis to provide statistical evidence for the significance of the study results with respect to the given number of cases. Based on the observed cross-sectional correlation of the NFRT with the BPS at baseline (N=52, r=0.35), the calculated power was 73 %.

## Discussion

The primary objective of the present study was to demonstrate whether NFRT measurement is associated with the BPS and RASS scores in critically ill, mechanically ventilated, and analgosedated patients who are unable to communicate. We also investigated whether the measured stimulus thresholds differ between patient groups and whether the NFRT measurement can be used to detect potential excessive analgesia.

### Association between the NFRT, the BPS and the RASS score

Critically ill, mechanically ventilated patients who are unable to communicate need pain assessment that is as accurate as possible. However, established pain assessment scales such as the BPS reach their limits in patients under profound analgosedation (RASS score ≤ -4) [7, 24]. As pain cannot essentially be measured during deep analgesia, one complementary approach is to assess nociceptive processes by measuring the NFRT stimulus threshold [21, 23]. In the present study, the NFRT was shown to be associated negatively with the depth of sedation. This is consistent with results obtained in patients under general anesthesia [14, 15, 18, 23]. The majority of the patients in the present study were profoundly analgosedated (RASS scorer ≤ -4). The NFRT measurement is also associated negatively with BPS scores in these patients.

Furthermore, the results indicate that patients with presumed absence of pain have higher stimulus thresholds than patients with mild pain. Thus, NFRT measurement could help to better assess patient analgesia, especially in patients where clinically established pain assessment scales reach their limits. However, this also implies that the method will only be truly beneficial in a select few patients.

### FRT measurement in patients with different medical specialties/diseases

There is a high risk of misinterpretation of analgesia by the treatment team in profoundly analgosedated patients. In a study by Whipple et al., a large proportion of the treatment team recorded freedom from pain in patients who retrospectively reported severe to extremely severe pain [25]. Therefore, special attention was paid to patients with RASS scores ≤ -4 and BPS scores of between 3 and 4 in the present study. Considering the various surgical procedures/diseases, we noted that different surgeries/diseases result in different stimulus thresholds despite comparable depths of sedation. This may be due to the fact that various surgeries/diseases cause different levels of nociception [25–29]. The different stimulus thresholds in patients with different diseases cannot be explained by the dosage of analgesics administered. This is in agreement with the results of a study by Dincklage et al. on “Monitoring of painful stimuli under anesthesia with remifentanil and propofol” [30]. The same concentration of remifentanil caused varying increases in NFRTs.

### Identifying excessive analgesia using NFRT measurement

Most patients in the present study with a RASS score ≤ -4 were excessively analgosedated. In the context of modern intensive care medicine and the credo “more analgesia, less sedation,“ such deep sedation can only be considered appropriate in exceptional cases [7–9, 31, 32]. However, a reduction of analgesia to RASS scores of between -3 to 0 seems to be favorable because it results in a lower incidence of delirium, improved weaning, etc. It is conceivable that in deeply sedated patients, the individual stimulus threshold at which the patient perceives pain can be detected by means of a gradual reduction of analgesics and sedatives and repetitive NFRT measurements. Titration of the analgesics used just above this particular stimulus threshold could be considered as adequate analgesia. Ideally, this approach would result in a reduction in the depth of sedation.

## Limitations

The study is subject to a number of limitations that need to be discussed.

### Limitations in the study design:

The chosen study design had to be modified to that of a pilot study due to its premature termination as a result of the SARS–CoV–2 pandemic. The reduction of the number of cases studied to half of the initially planned number does not allow for a statistically valid subgroup. The disparity observed between the two groups may have been due to the premature termination of the study, although the number of cases was balanced at the time of termination.

Because NFRT measurement is not conducted regularly in critically ill patients, Group B (BPS measurements only) was created as a control group. Group B is thus descriptive in nature and does not contribute towards answering the primary research questions. BPS scores of 3 and 4 were recorded for almost all patients in Group A. Due to the very small number of measurements with BPS scores ≥5, it remains unclear which NFRTs can be recorded in critically ill patients with severe to very severe pain. Due to the lack of blinding for the clinicians, the possibility that the NFRT measurements had an impact, albeit unintended, on the analgosedation regimen of the patients in Group A cannot be excluded with certainty.

Because the study was performed in a heterogeneous patient cohort of critically ill patients, it is not possible to extrapolate the results to internal medicine or cardiac surgery patients. The administration of analgesics and hypnotics must also be discussed as a major point of criticism. The study was conducted in a clinical practice. Therefore, some patients received remifentanil when the measurements were begun and sufentanil later on. This hampers the interpretation of the influence of the analgesics used on the measured NFRT stimulus thresholds considerably. Furthermore, the dosage of the analgesics and hypnotics administered was not adapted to each patient’s body weight.

### Limitations in the statistical analyses

Because of the repeated NFRT measurements performed for each patient, it is not possible to perform any simple correlation analyses. Thus, no correlation coefficients can be provided for the association between BPS, NFRT, and RASS. This complicates the interpretation of our results.

## Conclusions

NFRT measurement is useful in detecting potentially excessive analgesia in critically ill, analgosedated, mechanically ventilated patients who are unable to communicate. In particular, very deeply sedated patients in whom pain assessment using the BPS method indicates that only patients with slight pain or freedom of pain would benefit from NFRT measurement. In these patients, a gradual reduction of analgesics and sedatives to the specific NFRT above which the patient perceives no pain could lead to more appropriate analgesia with a decrease in sedatives. Further studies are needed to systematically examine the use of NFRT measurement in a heterogenous group of critically ill, mechanically ventilated patients who are unable to communicate.

## Supplementary Information

**Additional file 1.**

## Data Availability

The datasets used and/or analyzed during the current study are available from the corresponding author on reasonable request.

## Abbreviations

IASP: International Association for the study of pain
BPS: Behavioral Pain Scale
EMG: Electro Myography
ICU: Intensive Care Unit
LM: Linear Model
MLM: Mixed Linear Regression Modelling
NFR: Nociceptive Flexor Reflex
NFRT: Nociceptive Flexor Reflex Threshold
NRS: Numeric Rating Scale
RASS: Richmond Agitation Sedation Scale
SAPS II: Simplified Acute Physiology Score II
SOFA-Score: Sequential organ failure assessment score
TISS 28: Therapeutic Intervention Scoring System
