# Supplementary material for: Measurement of the nociceptive flexion reflex threshold in critically ill patients – a randomized observational pilot study
Source: BMC Anesthesiol. 2021 Nov 5;21:270. doi: 10.1186/s12871-021-01490-8 (PMC8569046; doi:10.1186/s12871-021-01490-8)
Supplement: Supplementary file 1 — Additional file 1. [file 12871_2021_1490_MOESM1_ESM.docx]

**Measurement of the nociceptive flexion reflex threshold in critically ill patients – a randomized observational pilot study**

Benedikt Schick^1🖂^, Benjamin Mayer^2^, Steffen Walter^3^, Sascha Gruss^3^, Ronald Stitz^1^, Pauline Stitz^1^, Eberhard Barth^1^

^1^Department of Anesthesiology and Intensive Care Medicine, University Hospital Ulm, Albert-Einstein-Allee 23, 89081, Ulm, Germany

^2^Ulm University, Institute of Epidemiology and Medical Biometry, Schwabstraße 13, 89075 Ulm, Germany

^3^Ulm University, Department of Medical Psychology, Frauensteige 6, 89075 Ulm, Germany

*🖂* corresponding author: benedikt-1.zujalovic@uni-ulm.de

**Figure Legends additional file**

**Table 1: Calculation of the influence of demographic factors on the NFRT using a univariate analysis.**

| **Independent variable** | **NFR-threshold** | | |
| --- | --- | --- | --- |
|  | Estimate | SE | P-value |
| **Age** | -0.38 | 0.37 | 0.299 |
| **Sex (male vs female)** | 12.56 | 11.44 | 0.278 |
| **Size** | 0.87 | 0.58 | 0.143 |
| **Weight** | 0.42 | 0.31 | 0.184 |
| **Length of stay in ICU** | 2.55 | 1.81 | 0.166 |

Univariate Linear model analyses to evaluate possible associations between NFRT measurement and the key baseline characteristics. The analysis refers to the variables listed in Table 1 "Patient characteristics" in the manuscript. SE = standard error, ICU = Intensive Care Unit.

**Table 2: Presentation of the results from Figure 5 in tabular form**

| **Specialty** | **BPS** | **NFRT_Median_ mA** | **IQR* mA** |
| --- | --- | --- | --- |
| Neurosurgery | 3 | 20.50 | 15.02 – 37.30 |
| Thoracic surgery | 3 | 62.00 | 16.30 – 114.00 |
| Respiratory failure | 3 | 37.40 | 17.80 – 87.00 |
| Abdominal surgery | 3 | 41.00 | 32.50 – 54.20 |
| Trauma surgery | 3 | 40.00 | 27.40 – 80.00 |
| Urology | 3 | 23.25 | 11.25 – 35.38 |
|  | | | |
| Neurosurgery | 4 | 13.75 | 4.00 – 28.00 |
| Thoracic surgery | 4 | 24.60 | 16.90 – 150.00 |
| Respiratory failure | 4 | 95.00 | 28.00 – 99.00 |
| Abdominal surgery | 4 | 36.90 | 26.82 – 47.50 |
| Trauma surgery | 4 | 16.45 | 3.75 – 28.55 |
| Urology | 4 | 24.95 | 4.03 – 72.35 |

The numerical results of the graphical representation of the main text are shown from Figure 4 (NFRTs versus BPS, related to the different disciplines) are shown above. Due to the small number of patients, an extensive statistical analysis of the results was not performed. BPS = Behavioral Pain Scale, NFRT = nociceptive flexion reflex threshold, IQR = Interquartile Range, mA = milliampere

**Table 3 Presentation of the Results of Figure 6 in tabular form**

| \| RASS \| \| NFRT_Median_ (IQR) \| P-value \| RASS \| \| NFRT_Median_ (IQR) \| P-value \| \| --- \| --- \| --- \| --- \| --- \| --- \| --- \| --- \| \| -5 \| -4 \| 59.40 (32.95 – 91.00)  29.00 (13.56 – 51.52) \| < 0.001 \| -2 \| -1 \| 30.50 (21.50 – 40.63)  17.90 (9.50 – 32.00) \| 0.002 \| \|  \| -3 \| 31.75 (16.88 – 44.92) \| < 0.001 \|  \| 0 \| 11.50 (7.05 – 14.80) \| < 0.001 \| \|  \| -2 \| 30.50 (21.50 – 40.63) \| < 0.001 \|  \| 1 \| 19.50 (14.10 – 24.90) \| 0.233 \| \|  \| -1 \| 17.90 (9.50 – 32.00) \| < 0.001 \|  \|  \|  \|  \| \|  \| 0 \| 11.50 (7.05 – 14.80) \| < 0.001 \|  \|  \|  \|  \| \|  \| 1 \| 19.50 (14.10 – 24.90) \| 0.032 \|  \|  \|  \|  \| \|  \| \| \| \|  \|  \|  \|  \| \| -4 \| -3 \| 29.00 (13.56 – 51.52)  31.75 (16.88 – 44.92) \| 0.354 \| -1 \| 0 \| 30.50 (21.50 – 40.63)  11.50 (7.05 – 14.80) \| 0.026 \| \|  \| -2 \| 30.50 (21.50 – 40.63) \| 0.737 \|  \| 1 \| 19.50 (14.10 – 24.90) \| 0.930 \| \|  \| -1 \| 17.90 (9.0 – 32.00) \| 0.033 \|  \|  \|  \|  \| \|  \| 0 \| 11.50 (7.05 – 14.80) \| 0.004 \|  \|  \|  \|  \| \|  \| 1 \| 19.50 (14.10 – 24.90) \| 0.528 \|  \|  \|  \|  \| \|  \| \| \| \|  \|  \|  \|  \| \| -3 \| -2 \| 31.75 (16.88 – 44.92)  30.50 (21.50 – 40.63) \| 1.000 \| 0 \| 1 \| 11.50 (7.05 – 14.80)  19.50 (14.10 – 24.90) \| 0.392 \| \|  \| -1 \| 17.90 (9.50 – 32.00) \| 0.002 \|  \|  \|  \|  \| \|  \| 0 \| 11.50 (7.05 – 14.80) \| < 0.001 \|  \|  \|  \|  \| \|  \| 1 \| 19.50 (14.10 – 24.90) \| 0.338 \|  \|  \|  \|  \| |
| --- | --- | --- | --- | --- | --- | --- | --- | --- | --- | --- | --- | --- | --- | --- | --- | --- | --- | --- | --- | --- | --- | --- | --- | --- | --- | --- | --- | --- | --- | --- | --- | --- | --- | --- | --- | --- | --- | --- | --- | --- | --- | --- | --- | --- | --- | --- | --- | --- | --- | --- | --- | --- | --- | --- | --- | --- | --- | --- | --- | --- | --- | --- | --- | --- | --- | --- | --- | --- | --- | --- | --- | --- | --- | --- | --- | --- | --- | --- | --- | --- | --- | --- | --- | --- | --- | --- | --- | --- | --- | --- | --- | --- | --- | --- | --- | --- | --- | --- | --- | --- | --- | --- | --- | --- | --- | --- | --- | --- | --- | --- | --- | --- | --- | --- | --- | --- | --- | --- | --- | --- | --- | --- | --- | --- | --- | --- | --- | --- | --- | --- | --- | --- | --- | --- | --- | --- | --- | --- | --- | --- | --- | --- | --- | --- |

Statistical analysis of the NFR threshold as a function of the assessed RASS values (Mann-Whitney rank sum test or Welch's t-test - RASS = Richmond Agitation Sedation Scale, NFRT = nociceptive flexion reflex threshold, IQR = Interquartile Range)
